# The Force of Uterine Contraction

**Published:** 1870-12

**Authors:** 


The Force of Uterine Contraction.—The extreme force of uterine contraction produces a pressure of 3,402 lbs. per square inch, which is equivalent to a pressure of 54·106 lbs, acting upon a circle of 9½ inches in diameter, which is assumed as the average area of the pelvic canal. The maximum force used to expel the fœtus, by both uterine and abdominal muscles combined, is estimated by Soulin, by forceps experiments made on the dead body, at 110.23 lbs., a result which is regarded by Dr. Duncan as too large. Dr. Duncan considers 80 lbs. as the maximum force ever employed in difficult cases. This would correspond with an hydrostaticol pressure inside the uterus of 5.05 lbs. per square inch, which is greater than the uterine muscles, unaided, are capable of producing.—*Dublin Quarterly Journal Medical Sciences.*

